# Adenylyl cyclase 6 plays a minor role in the mouse inner ear and retina

**DOI:** 10.1038/s41598-023-34361-y

**Published:** 2023-05-01

**Authors:** Pranav Dinesh Mathur, Junhuang Zou, Grace Neiswanger, Daniel Zhu, Yong Wang, Ali A. Almishaal, Deepti Vashist, H. Kirk Hammond, Albert H. Park, Jun Yang

**Affiliations:** 1 Department of Ophthalmology and Visual Sciences, Moran Eye Center, University of Utah, Salt Lake City, UT 84132 USA; 2 Department of Neurobiology, University of Utah, Salt Lake City, UT 84132 USA; 3 Division of Otolaryngology, Department of Surgery, University of Utah, Salt Lake City, UT 84132 USA; 4 Department of Communication Sciences and Disorders, University of Utah, Salt Lake City, UT 84112 USA; 5 Department of Speech-Language Pathology and Audiology, College of Applied Medical Sciences, University of Hail, Hail, 81451 Saudi Arabia; 6 Division of Cardiovascular Medicine, Department of Medicine, University of California, San Diego, VA San Diego Healthcare System, San Diego, CA 92161 USA; 7 Present Address: Vecprobio Inc., San Diego, CA 92126 USA

**Keywords:** Retina, Cochlea, Hair cell, Inner ear, Mechanisms of disease, Cell biology, Auditory system, Visual system

## Abstract

Adenylyl cyclase 6 (AC6) synthesizes second messenger cAMP in G protein-coupled receptor (GPCR) signaling. In cochlear hair cells, AC6 distribution relies on an adhesion GPCR, ADGRV1, which is associated with Usher syndrome (USH), a condition of combined hearing and vision loss. ADGRV1 is a component of the USH type 2 (USH2) protein complex in hair cells and photoreceptors. However, the role of AC6 in the inner ear and retina has not been explored. Here, we found that AC6 distribution in hair cells depends on the USH2 protein complex integrity. Several known AC6 regulators and effectors, which were previously reported to participate in ADGRV1 signaling in vitro, are localized to the stereociliary compartments that overlap with AC6 distribution in hair cells. In young AC6 knockout (*Adcy6*^*−/−*^) mice, the activity of cAMP-dependent protein kinase, but not Akt kinase, is altered in cochleas, while both kinases are normal in vestibular organs. Adult *Adcy6*^*−/−*^ mice however exhibit normal hearing function. AC6 is expressed in mouse retinas but rarely in photoreceptors. *Adcy6*^*−/−*^ mice have slightly enhanced photopic but normal scotopic vision. Therefore, AC6 may participate in the ADGRV1 signaling in hair cells but AC6 is not essential for cochlear and retinal development and maintenance.

## Introduction

Usher syndrome (USH) is the leading cause of inherited deaf-blindness with a prevalence as high as 1 in 6000 in the United States^1–4^. USH type 2 (USH2) is the most common of the three USH clinical types. USH2 manifests as congenital moderate to severe sensorineural hearing loss and retinitis pigmentosa, a condition caused by photoreceptor cell death. ADGRV1, USH2A, and whirlin proteins are encoded by the three known USH2 genes^5–7^. They interact with one another to form the ankle link complex at the tapering point of the stereociliary basal shaft in hair cells and the periciliary membrane complex between the inner and outer segments in photoreceptors^8–10^. The mechanism by which this USH2 complex functions in hair cells and photoreceptors has not been elucidated.

ADGRV1 has a seven transmembrane (7-TM) domain and is an adhesion G protein-coupled receptor (GPCR)^11,12^. In mammalian HEK293, PC12, and U251 cells, a short ADGRV1 fragment containing the 7-TM domain and the cytoplasmic C-terminal region constitutively couples with Gαi proteins and activates Gαi signaling^13^. In primary oligodendrocytes and mouse embryo fibroblasts, a mini ADGRV1 fragment containing 4 instead of 35 extracellular calcium-binding Calxβ domains couples with Gαs and Gαq proteins and activates cAMP-dependent protein kinase (PKA) and protein kinase C (PKC) δ/θ in response to extracellular calcium^14^. Furthermore, in an *Adgrv1* mutant mouse line (*Adgrv1*^*tm1Msat*^), adenylyl cyclase 6 (AC6) distribution and expression level are altered in cochlear stereocilia^9^. All these findings prompted us to propose that ADGRV1 activates Gαi, Gαs, and Gαq signaling under different physiological conditions and that AC6 may play a role downstream in the ADGRV1 signaling in hair cells and photoreceptors.

Adenylyl cyclases (ACs) are a group of enzymes that catalyze second messenger cAMP synthesis from ATP in GPCR signaling^15^. While all nine transmembrane ACs are stimulated by Gαs, only calmodulin-activated AC1 and Gαs-activated AC5 and AC6 are regulated by Gαi proteins, which are known to couple with and be activated by ADGRV1. Among the three ACs, AC1 is localized to the stereocilia in murine cochlear hair cells^16,17^. A nonsense mutation in *ADCY1*, the gene that encodes AC1, has been shown to cause inherited hearing loss in humans^17^, and knockdown of the zebrafish *adcy1b* gene diminishes the mechanotransduction in hair cells^17^, indicating the essential role of AC1 and cAMP in hair cells. In photoreceptors, AC1 is involved in the circadian secretion of melatonin^18,19^ and may regulate multiple proteins in the phototransduction cascade^20,21^. On the other hand, AC6 has been extensively studied in cardiac myocytes, where AC6 participates in the β-adrenergic receptor signaling pathway and activates Akt kinase (also known as protein kinase B) and phospholamban phosphorylation^22,23^. Mutations in the *ADCY6* gene were found to cause abnormal peripheral axon myelination and lethal arthrogryposis multiplex congenita in humans^24–26^. In the cochlea, reverse transcription-polymerase chain reaction (RT-PCR) and immunofluorescence studies have detected AC6 expression in hair cells, and the AC6 expression is affected by *Adgrv1* knockout^9^. However, the expression of AC6 in the retina and the function of AC6 in the inner ear and the retina have not been investigated.

In this study, we examined the relationship between AC6 and the ankle link complex as well as the expression of Gα, PKA, and Akt in inner ear hair cells. Unlike in humans, *Adcy6* mutations are not lethal in mice^23,27–29^. Using an *Adcy6*^*−/−*^ mouse model, we studied the auditory function of AC6 by auditory brainstem response (ABR) and distortion production otoacoustic emission (DPOAE) tests. We further explored the expression and function of AC6 in retinal photoreceptors. Our findings demonstrate that AC6 distribution in stereocilia relies on the ankle link complex integrity and that AC6 may function in the ADGRV1 signaling pathway in hair cells. AC6, however, is dispensable for normal hearing in mice. Additionally, AC6 is expressed mainly in the inner retina but not in photoreceptors and plays a minor role in photopic vision. Therefore, AC6 likely contributes insignificantly to the pathogenesis of hearing and vision loss caused by USH2 gene mutations.

## Results

### AC6 is localized to the basal portion of stereocilia in inner ear hair cells, which is maintained by the ankle link complex integrity

**Figure 1:**
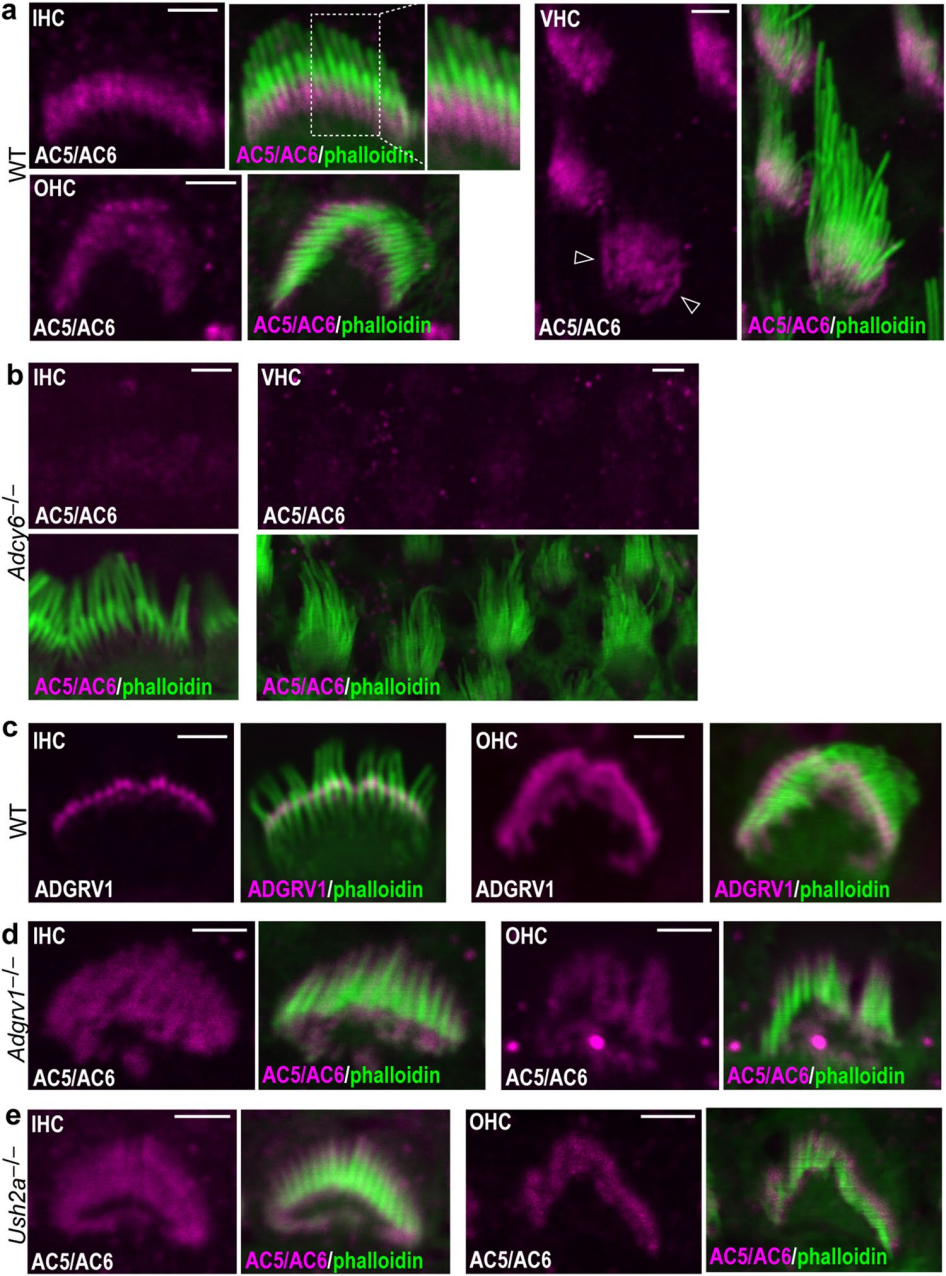
AC6 stereociliary localization in wild-type, *Adgrv1*^*−/−*^, and *Ush2a*^*−/−*^ inner ear hair cells. (**a**) AC6 (magenta) is localized to the basal portion of stereocilia stained by phalloidin (green) in a wild-type cochlear inner hair cell (IHC) and outer hair cell (OHC) as well as in wild-type vestibular hair cells (VHCs) at P4. The framed region in the merged view of the IHC is enlarged and shown to the right. Arrows in the VHC view point to the AC6 signal on the plasma membrane of VHC cell body. (**b**) AC6 immunostaining signal is lost in an *adcy6*^*−/−*^ IHC and VHCs at P4. (**c**) ADGRV1 (magenta) labels the ankle link complex in a wild-type cochlear IHC and OHC at P4. (**d** and **e**) The distribution of AC6 signal is extended to the entire stereocilia in the IHCs and OHCs of *Adgrv1*^*−/−*^ (**d**) and *Ush2a*^*−/−*^ (**e**) mice at P4. Note that the stereocilia in *Adgrv1*^*−/−*^ and *Ush2a*^*−/−*^ cochlear hair cells are shown in a fallen orientation. The merged images are the corresponding view of the magenta-colored images. Scale bars: 2 μm.

AC6 localization in inner ear hair cells was investigated by immunostaining whole mount mouse cochleas and vestibular organs using an antibody that recognized both AC5 and AC6 proteins. Immunoreactivity was detected at the basal portion of stereocilia in wild-type cochlear inner hair cells (IHCs), outer hair cells (OHCs), and vestibular hair cells (VHCs) at postnatal day 4 (P4) (Fig. 1a and Supplementary Fig. S1a), but the immunoreactivity was absent in *Adcy6*^*−/−*^ cochlear and vestibular hair cells at the same age (Fig. 1b and Supplementary Fig. S1b). This result indicates that AC5 protein expression in inner ear hair cells was undetectable by the AC5/AC6 antibody, consistent with a previous report demonstrating extremely low expression of AC5 mRNA in mouse cochlear hair cells by single-cell RT-PCR^9^. Therefore, the signals detected by the AC5/AC6 antibody in mouse inner ear hair cells were derived from the AC6 protein, and AC6 is present in the hair cell stereociliary basal portion in the inner ear.

Because our antibodies against the AC6 and USH2 proteins were generated from the same species, we could not perform double immunostaining experiments to directly investigate whether AC6 distribution overlaps with the ankle link complex in stereocilia. Instead, we compared the AC6 and ADGRV1 single immunostaining signals in stereocilia (Fig. 1a,c and Supplementary Fig. S1a,c). AC6 appeared to be in proximity to the ankle link complex at the stereociliary base. We then examined AC6 distribution in *Adgrv1*^*−/−*^ and *Ush2a*^*−/−*^ cochleas, where the ankle link complex is disrupted^9,30^. Immunostaining of *Adgrv1*^*−/−*^ and *Ush2a*^*−/−*^ cochleas at P4 showed that AC6 distribution extended to the entire stereocilia (Fig. 1d,e and Supplementary Fig. S1d,e). This change was consistent with a previous observation in cochlear hair cells of another *Adgrv1* knockout mouse line, *adgrv1*^*tm1Msat*^, at a later time point (P7)^9^. In summary, our results demonstrated that AC6 is located at the basal portion of stereocilia close to the ankle link complex region in inner ear hair cells and that this distribution of AC6 requires the integrity of the ankle link complex.

### Several proteins in the AC6 and ADGRV1 signaling pathway are located in the stereociliary basal portion, among which PKA activity is reduced in *Adcy6*^*−/−*^ cochleas

**Figure 2:**
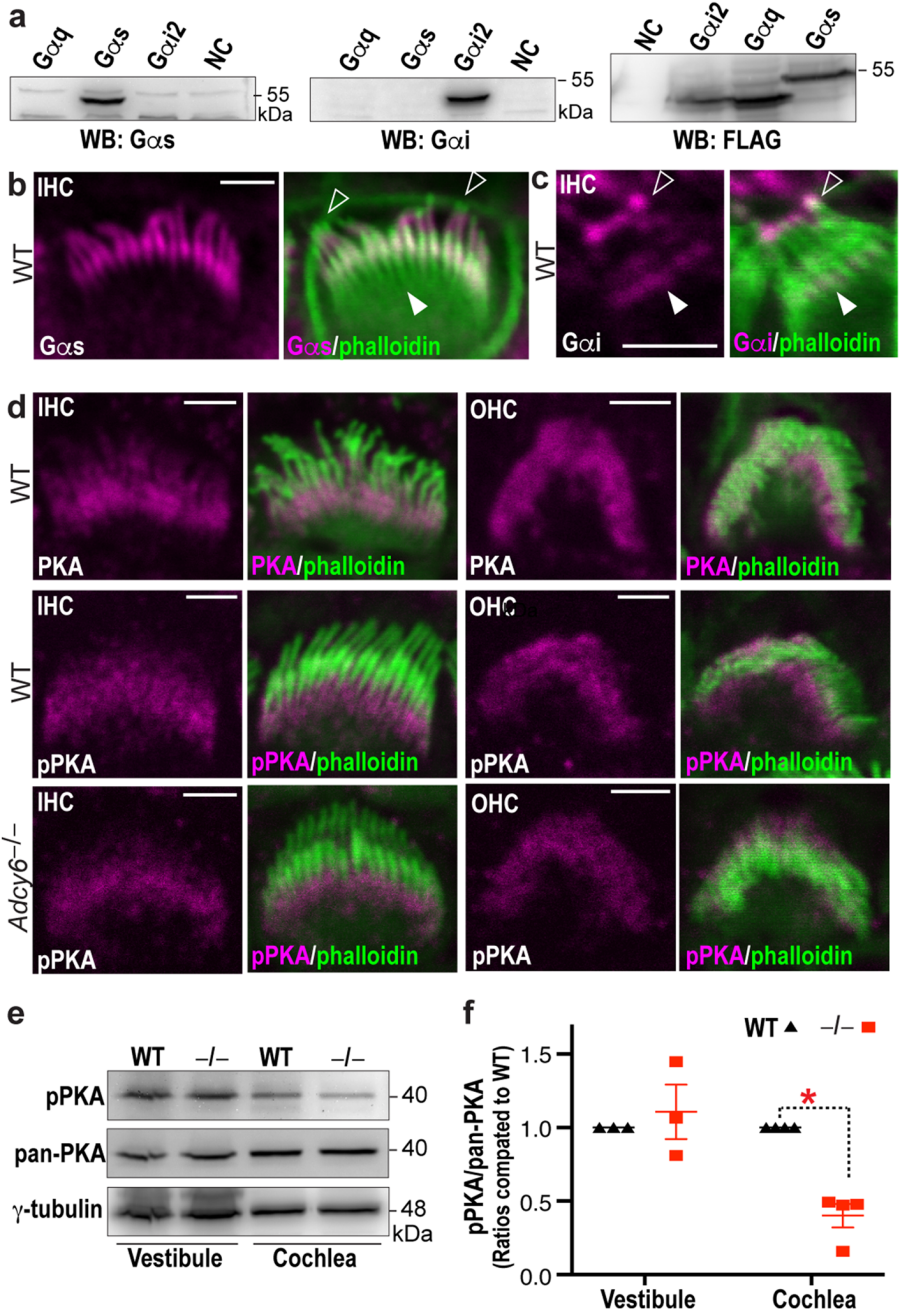
Gα and PKA localization in cochlear stereocilia and PKA activity reduction in *Adcy6*^*−/−*^ cochleas. (**a**) The specificity of Gαs and Gαi antibodies was verified by immunoblot analysis using their corresponding FLAG-tagged recombinant proteins transfected in HEK293 cells. The immunoblot detected by a FLAG antibody (right) was used to confirm the transfected Gα protein expressions. NC: untransfected HEK293 cell lysate. The original uncropped blot images are presented in Supplementary Fig. S4. (**b**) Immunostaining shows that Gαs protein (magenta) is located along the entire stereocilia (green) except the tip (arrows) and base (filled arrow) in a wild-type cochlear hair cell at P4. (**c**) Immunostaining shows that Gαi protein (magenta) is located at the very tip (arrow) and around the ankle link complex region (filled arrow) in wild-type cochlear stereocilia at P4. (**d**) Immunostaining shows that pan-PKA Cα1 (PKA, magenta, upper row) and phospho-PKA Cα1 (pPKA, magenta, middle row) are located at the basal portion of stereocilia in wild-type IHCs (left) and OHCs (right). The distribution of phospho-PKA Cα1 subunit in *Adcy6*^*−/−*^ cochlear hair cells (lower row) is similar to that in wild-type cochlear hair cells (middle row). (**b**–**d**) The merged images correspond to the single-channel magenta images. Scale bars: 2 µm. (**e**) Immunoblot analysis shows that pan-PKA Cα1 expression levels in *Adcy6*^*−/−*^ (^*−/−*^) cochleas and vestibular organs are similar to those in wild-type (WT) cochleas and vestibular organs. Phospho-PKA Cα1 subunit level is reduced by ~ 50% in *Adcy6*^*−/−*^ cochleas but remains normal in *Adcy6*^*−/−*^ vestibular organs. The blot images are representative images from three to four biologically independent experiments. γ-tubulin was used as a loading control. The original uncropped blot images are presented in Supplementary Fig. S4. (**f**) Quantification of the phospho-PKA Cα1 immunoblot signal intensities from three to four biologically independent experiments. The data are presented as ratios of phospho-PKA Cα1 to pan-PKA Cα1 and are normalized by corresponding wild-type ratios on the same blots. *, P value = 0.029 (Mann–Whitney test). Each point represents an individual biological sample pooled from at least 4 pups. Mean and SEM (standard error of the mean) are shown.

AC6 functions in GPCR signaling^15,31^, and ADGRV1 is an adhesion GPCR^11,12^. The similar distribution of AC6 with ADGRV1 and the dependence of AC6 distribution on ADGRV1, as described above, supported the notion that AC6 may be a downstream target of ADGRV1. We thus studied several proteins that could be in the AC6 and ADGRV1 signaling pathway in inner ear hair cells. Gαs and Gαi proteins stimulate and inhibit AC6 activity, respectively^32^. Both Gαs and Gαi couple with and are activated by ADGRV1^13,14^. Thus, we performed immunostaining of wild-type cochleas for these two proteins using antibodies whose specificity was confirmed by immunoblotting of recombinant Gαs and Gαi proteins in cultured cells (Fig. 2a). In cochlear hair cells at P4, Gαs was located along the entire stereocilia except at the very base or tip (Fig. 2b and Supplementary Fig. S2a). This signal pattern was more obvious in IHCs than in OHCs, because of the better morphology of IHC bundles than OHC bundles in the immunostaining images. Gαi immunoreactivity was seen at the stereociliary tip, as reported previously^33^, but weak Gαi immunoreactivity was also seen at a position similar to the ankle link complex in stereocilia (Fig. 2c and Supplementary Fig. S2b). Again, this signal pattern was more clearly observed in IHCs than OHCs. In summary, Gαs and Gαi proteins likely localize in proximity to AC6 and ADGRV1 in stereocilia.

PKA is activated by cAMP generated by AC6 and is also a downstream effector of ADGRV1^14,23^. The PKA holoenzyme consists of two regulatory subunits and two catalytic subunits. The major PKA catalytic subunit is catalytic subunit α isoform 1 (Cα1), which is expressed in most mammalian tissues^34^. The phosphorylation of Cα1 at threonine 198 (Thr198) is essential for PKA activation and has been used as an indicator for PKA activity^34^. We thus investigated total and activated PKA in the inner ear using an antibody against pan-PKA Cα1 subunit and an antibody against phosphorylated Thr198 of PKA Cα1, respectively. Immunostaining showed that pan-PKA and phosphorylated PKA had similar distributions in P4 wild-type cochlear IHCs and OHCs (Fig. 2d and Supplementary Fig. S2c,d). They were both localized to the basal portion of stereocilia, similar to the distribution of AC6 (Fig. 1a). In the IHCs and OHCs of *Adcy6*^*−/−*^ mice at P4, the distribution of phosphorylated PKA appeared normal (Fig. 2d and Supplementary Fig. S2e). Immunoblot analysis on three or four independent sets of biological samples consistently found that pan-PKA expression in cochleas and vestibular organs and phosphorylated PKA level in vestibular organs were not significantly altered in *Adcy6*^*−/−*^ mice, but phosphorylated PKA level in *Adcy6*^*−/−*^ cochleas was reduced by ~ 50% (P value = 0.029, Mann–Whitney test, Fig. 2e,f). Therefore, the similar distribution of PKA and AC6 in cochlear stereociliary basal shafts and the reduction of PKA activity in *Adcy6*^*−/−*^ cochleas suggest that PKA is likely activated by AC6 in the ADGRV1/AC6 signaling pathway in cochlear hair cells.

### Akt phosphorylation is normal in *Adcy6*^*−/−*^ inner ears

**Figure 3:**
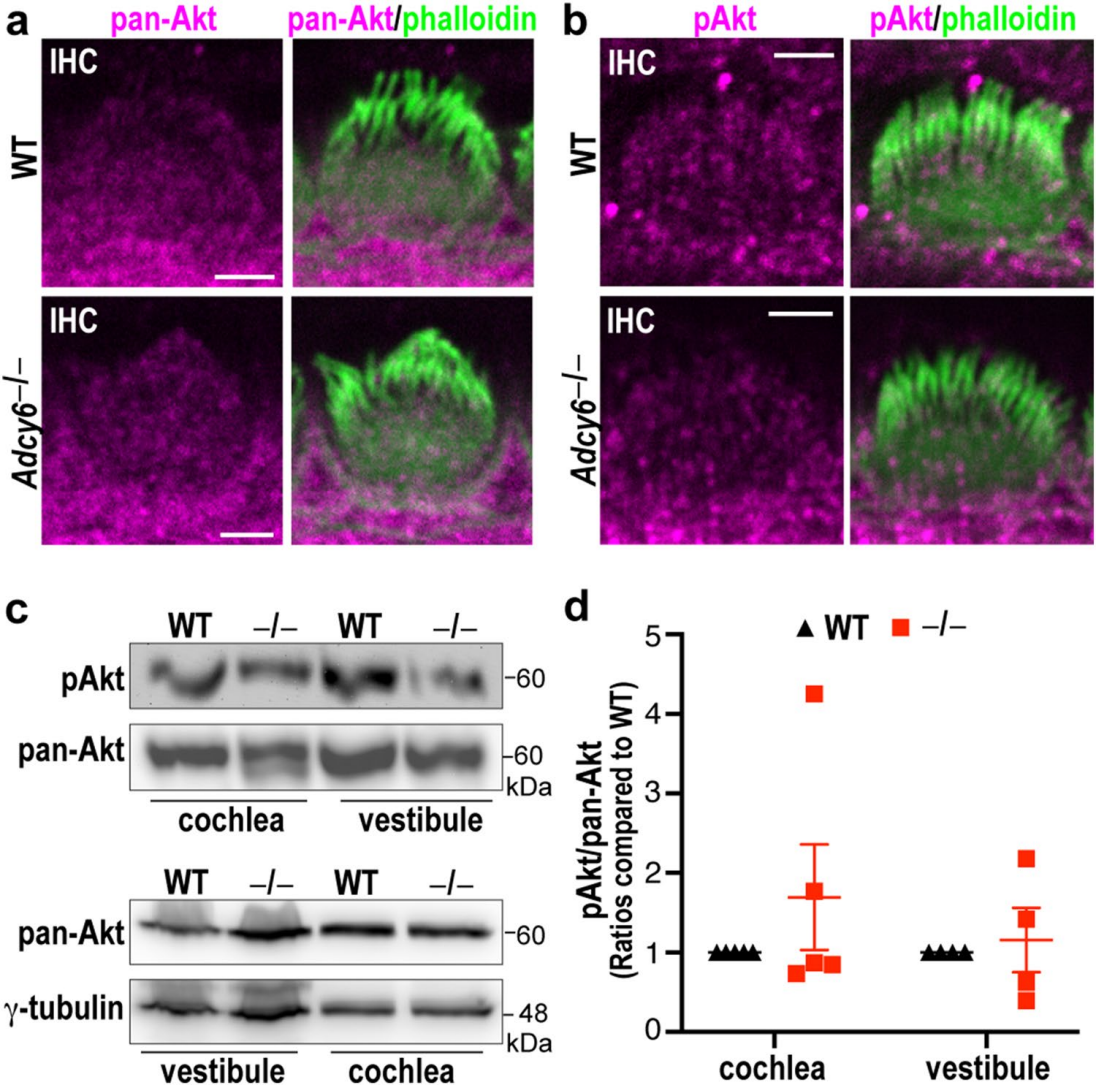
Normal Akt localization, expression, and phosphorylation in *Adcy6*^*−/−*^ inner ears. (**a** and **b**) Immunoreactivities of pan-Akt (magenta, **a**) and phospho-Akt (pAkt, magenta, **b**) are detected in the stereociliary bundle (green) and cell body of cochlear hair cells as well as surrounding cells in wild-type (upper row) and *Adcy6*^*−/−*^ (lower row) littermate mice at P4. The merged images (right) correspond to the single-channel magenta images (left). Scale bars: 2 µm. (**c**) Immunoblot analysis shows that pan-Akt (lower) and phospho-Akt (upper) levels in cochleas and vestibular organs are similar between *Adcy6*^*−/−*^ (^*−/−*^) and wild-type (WT) mice at P4. The blot images are representative images from four to five biologically independent experiments. The original uncropped blot images are presented in Supplementary Fig. S4. (**d**) Quantification of the phospho-Akt immunoblot signal intensities from four to five biologically independent experiments. The data are presented as ratios of phospho-Akt to pan-Akt and are normalized by corresponding wild-type ratios. Each point represents an individual biological sample pooled from at least 4 pups. Mean and SEM are shown.

Akt phosphorylation at serine 473 and threonine 308 was previously discovered as an AC6 signaling downstream event in primary neonatal rat cardiac myocytes^22^. Akt is a serine/threonine kinase in the phosphoinositide-3-kinase signaling pathway. It has been demonstrated that Akt1, as well as its two other Akt isoforms, is important for hearing function in mice^35^. Additionally, *Akt1*^*−/−*^ mice are sensitive to noise-induced hearing loss^36^. Based on these previous findings, we investigated whether AC6 functioned through Akt phosphorylation in inner ear hair cells. Immunostaining of wild-type and *Adcy6*^*−/−*^ cochleas at P4 using antibodies recognizing all three Akt isoforms and phosphorylated Ser473 of all three Akt isoforms showed that pan-Akt and phosphorylated Akt (pAkt) were present in almost all cells including hair cells. The staining signal pattern of pAkt was consistent with that in a previous report using the same antibody^36^. In hair cells, pan-Akt and pAkt were localized to the stereociliary bundles as well as the cell body (Fig. 3a,b and Supplementary Fig. S3). The distributions of pan-Akt and pAkt in *Adcy6*^*−/−*^ cochleas were similar to those in wild-type cochleas (Fig. 3a,b and Supplementary Fig. S3). Immunoblot analysis on five independent sets of cochlear samples and four independent sets of vestibular samples showed that, although pAkt expression had a large variation, the pAkt and pan-Akt expression levels in cochlear and vestibular organs were not statistically different between *Adcy6*^*−/−*^ and wild-type mice (Fig. 3c,d). Therefore, unlike in cardiac myocytes, Akt phosphorylation in the inner ear was not affected by AC6 expression, suggesting that the hearing and vestibular function of AC6 is unlikely to be mediated by Akt signaling.

### *Adcy6*^*−/−*^ mice exhibit normal hearing thresholds

**Figure 4:**
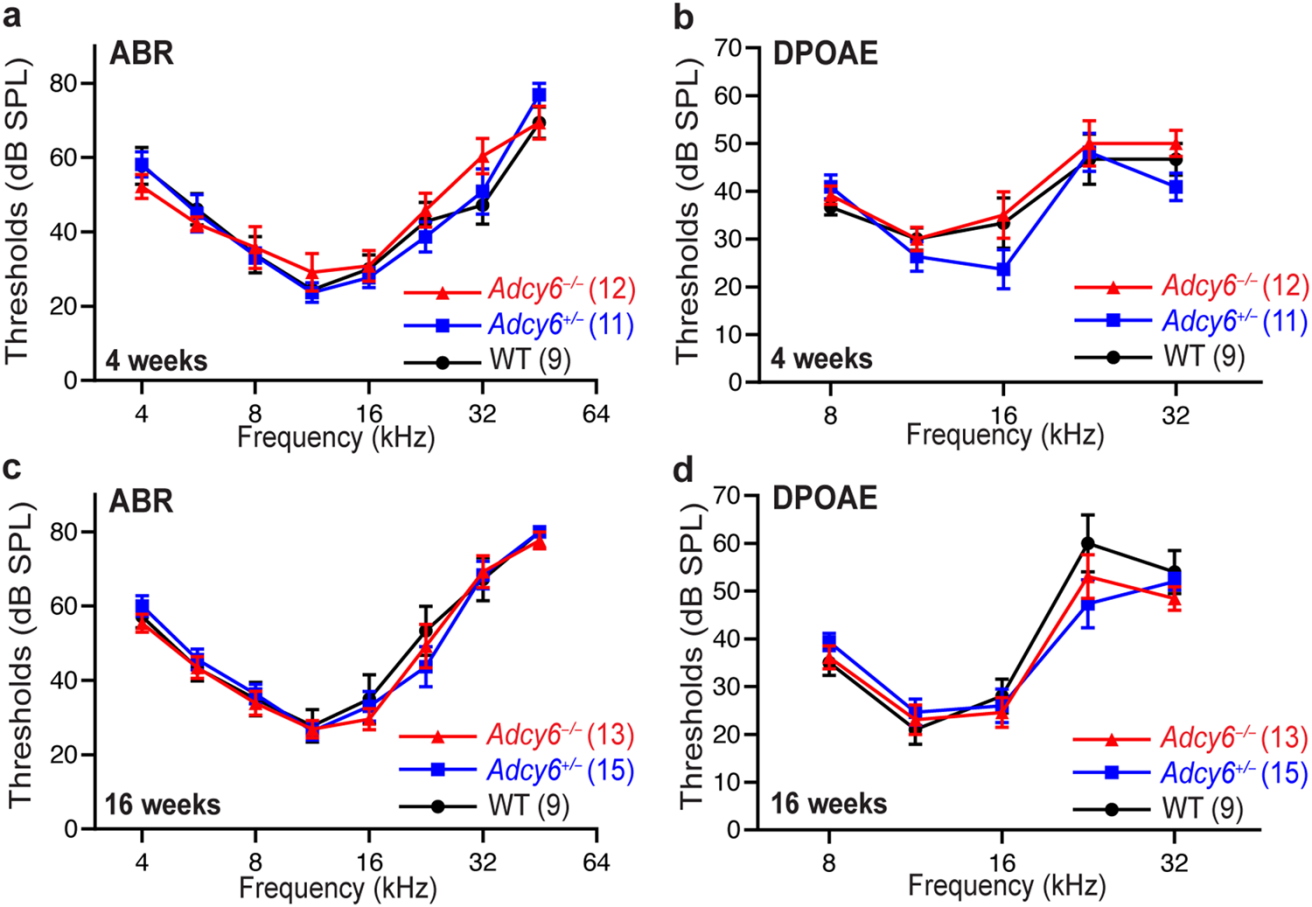
*Adcy6* mutant mice have normal hearing thresholds. (**a** and **b**) ABR (**a**) and DPOAE (**b**) tests did not detect hearing loss phenotype in *Adcy6*^*−/−*^ mice, compared with *Adcy6*^+*/−*^ and wild-type littermate mice at 4 weeks of age. (**c** and **d**) ABR (**c**) and DPOAE (**d**) thresholds in *Adcy6*^*−/−*^ mice are comparable to those of *Adcy6*^+*/−*^ and wild-type littermate mice at 16 weeks of age. (**a**–**d**) Repeated measures two-way ANOVA or mixed effects analysis was conducted with genotype as a between-subject variable and tone frequency as a within-subject variable. The P values for genotype are all above 0.05. The numbers of animals tested are shown in parentheses following the genotype in the legends. Data are shown as mean ± SEM.

To investigate the role of AC6 in the function of the inner ear, we assessed the hearing in young and mature *Adcy6*^*−/−*^ mice by ABR and DPOAE tests. *Adcy6*^*−/−*^ mice showed ABR thresholds at the tone frequencies from 4 to 45 kHz and DPOAE thresholds at the tone frequencies from 8 to 32 kHz, similar to those of their wild-type and *Adcy6*^+*/−*^ littermates at 4 and 16 weeks of age (Fig. 4). Therefore, *Adcy6*^*−/−*^ mice exhibited normal hearing function and did not show progressive hearing loss up to 16 weeks of age.

### AC6 is mainly expressed in mouse inner retinal cells

**Figure 5:**
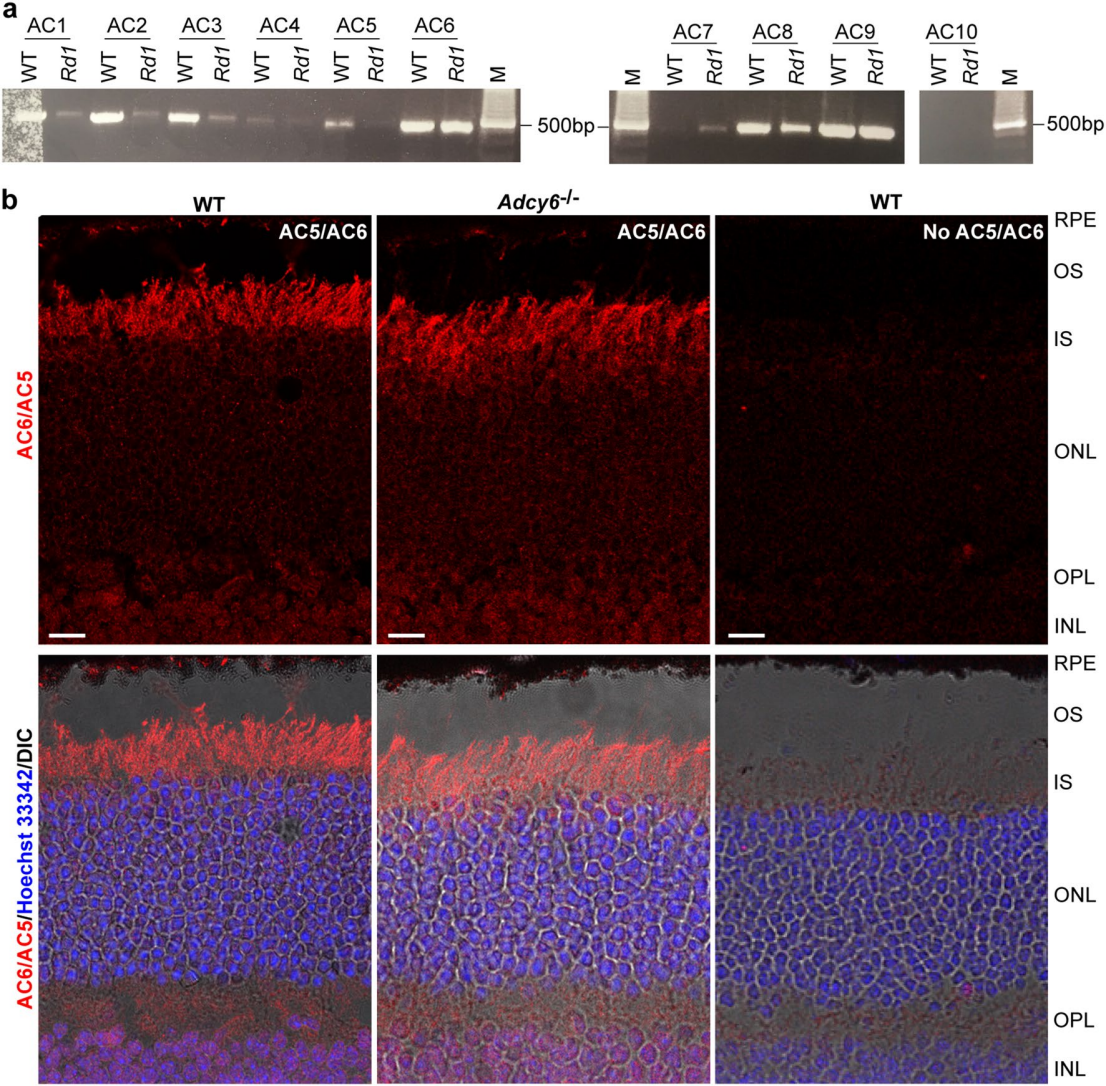
Expression of adenylyl cyclase genes in mouse retinas. (**a**) RT-PCR analysis of AC1-AC10 mRNA expressions in wild-type and *Rd1* mouse retinas at 2 months of age. M, DNA ladder. The original gel is presented in Supplementary Fig. S4. (**b**) Similar immunostaining signals from the AC5/AC6 antibody in adult wild-type (left) and *Adcy6*^*−/−*^ (middle) mouse retinas. Compared with the negative control immunostaining without the primary antibody (right), the signals are present in the photoreceptor cell body, but not outer segment. The signals are also present in the inner retina. RPE, retinal pigment epithelium; OS, outer segment; IS, inner segment; ONL, outer nuclear layer; OPL, outer plexiform layer; INL, inner nuclear layer; Hoechst 33,342, nuclear dye; DIC, differential interference contrast; scale bar: 10 µm.

Because AC6 distribution depended on the integrity of the ankle link complex in hair cells (Fig. 1d,e) and the ankle link complex has a similar multiprotein complex, the periciliary membrane complex, in photoreceptors^9,10^, we decided to study AC6 in the retina as well. First, we investigated AC1 to AC10 expression in photoreceptors and retinas by RT-PCR. *Rd1* mice at 2 months of age have been shown to lose almost all rod photoreceptors and the majority of cone photoreceptors^37^. We thus compared AC mRNA expressions between wild-type and *Rd1* retinas at this age (Fig. 5a). More AC1, AC2, AC3, AC4, and AC8 mRNAs were amplified from wild-type retinas than *Rd1* retinas, indicating that these AC genes were expressed in photoreceptors as well as other retinal cells. AC5 mRNA was mainly amplified from wild-type retinas, indicating that this AC gene was mostly expressed in photoreceptors. AC6 and AC9 mRNAs were amplified similarly from wild-type and *Rd1* retinas, suggesting that these two AC genes were mainly expressed in retinal cells other than photoreceptors. AC7 and AC10 mRNAs were not amplified in wild-type retinas, but for an unknown reason, AC7 amplification was increased in *Rd1* retinas. Immunostaining using the AC5/AC6 antibody detected signals in the photoreceptor cell body, but not the outer segment (Fig. 5b). Similar signal patterns and intensities were found in adult wild-type and *Adcy6*^*−/−*^ retinas (Fig. 5b), indicating that the immunoreactivity from the AC5/AC6 antibody in the retina, especially in the photoreceptors, is mainly derived from the AC5 protein and the AC6 protein is rarely expressed in photoreceptors.

### Photopic but not scotopic vision is slightly enhanced in *Adcy6*^*−/−*^ mice

**Figure 6:**
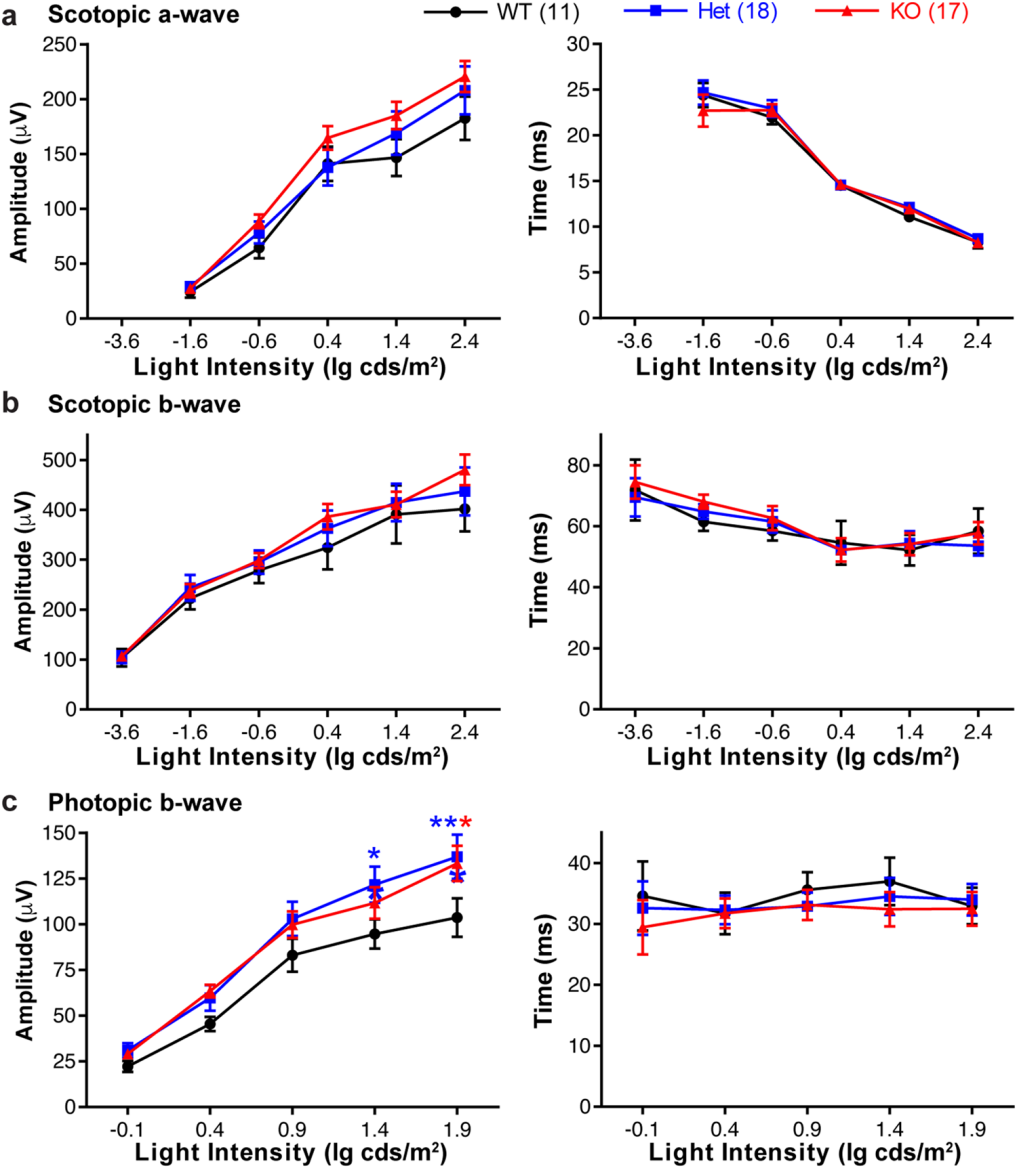
*Adcy6*^*−/−*^ mice have a slightly enhanced photopic ERG response. (**a**) The scotopic ERG a-wave amplitude (left) and implicit time (right) in *Adcy6*^*−/−*^ mice are comparable to those of *Adcy6*^+*/−*^ and wild-type littermate mice at 16 weeks of age. (**b**) The scotopic ERG b-wave amplitude (left) and implicit time (right) in *Adcy6*^*−/−*^ mice are normal, compared with *Adcy6*^+*/−*^ and wild-type littermate mice at 16 weeks of age. (**c**) The photopic ERG b-wave amplitude (left), but not implicit time (right), in *Adcy6*^*−/−*^ and *Adcy6*^+*/−*^ mice is slightly increased at light intensities of 1.4 and/or 1.9 lg cds/m^2^, compared with wild-type littermate mice at 16 weeks of age. Repeated measures analysis was conducted using the mixed effects model with genotype as a between-subject variable and light intensity as a within-subject variable. The P values for genotype are 0.378 and 0.690 (scotopic a-wave amplitude and implicit time, respectively, **a**); 0.713 and 0.823 (scotopic b-wave amplitude and implicit time, respectively, **b**); and 0.071 and 0.819 (photopic b-wave amplitude and implicit time, respectively, **c**). * and **, adjusted P value < 0.05 and 0.01, respectively (Dunnett’s multiple comparisons test to compare *Adcy6*^+*/−*^ and *Adcy6*^*−/−*^ data with wild-type data). The genotype legend on the top right applies to all panels. The numbers of animals tested are shown in parentheses following the genotype in the legend. All data are shown as mean ± SEM.

Electroretinogram (ERG) was conducted in wild-type, *Adcy6*^+*/−*^, and *Adcy6*^*−/−*^ littermate mice at 16 weeks of age. Although the statistical mixed-effects model did not reveal a significant difference in scotopic and photopic ERG amplitudes and implicit times among the three genotype groups, Dunnett’s multiple comparisons test found that, while the amplitudes and implicit times of scotopic a-wave and b-wave and the implicit time of photopic b-wave were the same among the three genotype groups, the photopic b-wave amplitude of *Adcy6*^*−/−*^ and *Adcy6*^+*/−*^ mice was statistically larger than that of wild-type littermates at high light intensities (1.4 and 1.9 lg cds/m^2^, Fig. 6). Taken together, our results showed that AC5 but not AC6 is abundant in photoreceptors and that *Adcy6*^*−/−*^ and *Adcy6*^+*/−*^ mice have a slightly enhanced photopic but not scotopic vision. Because scotopic and photopic ERGs detect the function of rod and cone visual pathways, respectively, AC6 is probably involved in the cone visual pathway that occurs in the inner retina.

## Discussion

In this study, we verified the distribution of AC6 in the stereociliary basal portion of cochlear hair cells and its dependence on ADGRV1 expression. We further extended the distribution of AC6 in the stereociliary basal portion to vestibular hair cells and the dependence of this distribution on ankle link complex integrity. Together with the previously reported in vitro activation of Gα proteins by ADGRV1^13,14^, the in vivo AC6 expression and distribution alterations in *Adgrv1* mutant hair cells^9^, and the activation of PKA by AC6 after Gα stimulation^15^, our findings of the distribution and/or expression of Gαs, Gαi, PKA, and Akt in inner ear hair cells suggest that AC6 might function in the ADGRV1-Gα-AC-PKA pathway and that Akt is not the downstream target of this pathway. In photoreceptors, AC5, the closest AC6 paralog, is more abundantly expressed than AC6. Knockout of *Adcy6* in mice slightly enhances retinal photopic ERG responses but has no effect on retinal scotopic ERG responses or auditory ABR or DPOAE thresholds. Our report is the first to investigate the AC6 expression in the retina and the AC6 physiological function in both the cochlea and the retina.

AC6 was previously shown to distribute along the entire stereocilia in mouse cochleas before P1. From P3 onward, AC6 moves to the basal portion of stereocilia^9^. Our finding of AC6 distribution along the entire stereocilia in *Adgrv1*^*−/−*^ and *Ush2a*^*−/−*^ mice at P5 is unlikely due to a developmental delay, because the same abnormal AC6 distribution was also observed in another *Adgrv1* null mouse at a later time point P7^9^. The time point when AC6 distribution switches is coincident with the ankle link complex emergence during development^38^. Whirlin in the ankle link complex is a scaffold protein^9,39^. This protein interacts with membrane-associated guanylate kinase (MAGUK) proteins p55 and calcium/calmodulin-dependent protein kinase (CASK)^40,41^, and p55 and CASK proteins interact with 4.1R and 4.1N proteins^42–44^. In cochlear hair cells, p55, CASK, 4.1R, and 4.1N proteins are located in stereocilia^40^. Recently, the AC6 N-terminus was discovered to interact with 4.1G protein^45^. Therefore, it is possible that the ankle link complex restricts the distribution of AC6 to the basal portion of stereocilia through an indirect association between whirlin and AC6, which is mediated by their direct interactions with MAGUK and 4.1 proteins.

Most adhesion GPCRs studied so far undergo autoproteolysis at the GPCR-autoproteolysis inducing domain. The resultant C-terminal fragment harbors the 7-TM domain and usually exhibits constitutive GPCR activity, while the N-terminal fragment regulates the constitutive activity through autoproteolysis^11,12^. Previous studies in cultured cells showed that the autoproteolyzed ADGRV1 C-terminal fragment has constitutive Gαi-coupling activity^13^ and that another C-terminal ADGRV1 fragment containing a small N-terminal portion is stimulated by extracellular calcium and activates Gαs-cAMP-PKA-CREB and Gαq-PKC δ/θ signaling pathways simultaneously^14^. Although it is currently unclear how the three Gα signaling pathways coordinate in different physiological conditions, these ADGRV1 signaling pathways could all occur in inner ear hair cells. In these cells, ADGRV1 is localized at the stereociliary ankle link complex^9,30,46,47^, while some ADGRV1 short variants without the signal transduction 7-TM domain might be at the synapse regions^48,49^. Therefore, the three ADGRV1 signaling pathways could occur at the stereociliary ankle link complex but not the synapse. We found that AC6, Gαs, Gαi, and PKA Cα1 subunit are present in the basal portion of cochlear stereocilia and that their distributions are presumed to overlap partially with that of ADGRV1. Furthermore, the expression of AC6 in the cochlea is responsible for the activation of ~ 50% PKA. These findings suggest that the ADGRV1-Gαs/ Gαi-AC6-PKA pathway may exist at the ankle link complex in cochlear hair cells. However, our study has limitations with regard to pinpointing the targets regulated by AC6 signaling at the ankle link complex. First, immunoblot analysis of PKA and phospho-PKA expression in inner ear tissues cannot represent the PKA activity change specifically at the ankle link complex in stereocilia, because PKA is expressed in many other cells in the tissues. Second, there are two main mammalian PKA catalytic subunit isoforms, Cα and Cβ, and each isoform has multiple splice variants^34^. Our study only examined the expression and phosphorylation of PKA Cα1 subunit in *Adcy6*^*−/−*^ inner ears. Therefore, the details of the AC6 signaling pathway and the mechanism by which AC6 functions with ADGRV1 in hair cells need to be further investigated.

AC1 is another predominant AC protein in the cochlea. This protein is located in the stereocilia, cell body, and nucleus of hair cells^16,17^. The hearing loss caused by an *ADCY1* mutation in humans and the abnormal mechanotransduction caused by *adcy1b* knockdown in zebrafish neuromast hair cells^17^ indicate an indispensable role of AC1 in hearing function. However, the exact signaling pathway of AC1 in hair cells has not been elucidated. AC1 is also regulated by Gαs and Gαi proteins^15^. The conserved motif in the AC1 C2 domain is very similar to that in AC6 (80%)^17^. Therefore, the functions of AC1 and AC6 may be redundant in hair cells, to some extent, which could explain the observed normal hearing in *Adcy6*^*−/−*^ mice. Another reason for the normal hearing in *Adcy6*^*−/−*^ mice is that the potential ADGRV1-Gαq-PKC δ/θ signaling pathway may still function and compensate for the AC6 loss in *Adcy6*^*−/−*^ hair cells. However, it is likely that *Adcy6*^*−/−*^ mice may have slight hearing impairment when challenged by aging or noise insult.

AC6 and AC5 are the closest paralogs with similar amino acid sequences in the AC protein family. Both are stimulated by Gαs and Gβγ and inhibited by PKA and calcium. Their regulations by Gαi and PKC are not exactly the same^15^. In the retina, we showed that AC5 mRNA is mainly expressed in photoreceptors by RT-PCR and the AC5 protein is present in the photoreceptor cell body by immunostaining of *Adcy6*^*−/−*^ retinas using the AC5/AC6 antibody. On the contrary, AC6 mRNA is much more abundant in other retinal cells than photoreceptors, which is consistent with the normal scotopic ERG a-wave observed in *Adcy6*^*−/−*^ mice, an indicator of normal rod photoreceptor function. The slight enhancement of photopic ERG b-waves in *Adcy6*^*−/−*^ and *Adcy6*^+*/−*^ mice suggests that AC6 may play a role in the visual pathway downstream of cone photoreceptors and that this pathway is sensitive to the expression level of AC6 in inner retinal cells. However, the exact role of AC6 and its exact functional cellular and subcellular locations along the cone-mediated visual pathway need to be further elucidated. Based on our findings, we propose that AC6 plays a minor role in the retina and may not participate in the ADGRV1 signaling pathway in photoreceptors, although we do not exclude the possibility that AC6 may be important for vision at an old age or expressed and function differently in mouse and human photoreceptors.

Four *ADCY6* mutations have been shown to cause lethal arthrogryposis multiplex congenita in humans^24–26^. Two of them are homozygous missense mutations (Y992C and R1116C), and the other two are compound heterozygous missense and splice site mutations (E1003K and c.1535 + 1G > A). Homology modeling suggests that the residues affected by these missense mutations are positioned at the interface between the AC6 C2 domain and Gαs, the interface between the AC6 C1 and C2 domains, and the interface between AC6 and its ATP substrate^24^. Therefore, these missense mutations are predicted to affect AC6 activity to synthesize cAMP, which is crucial for muscle, joint, and nervous system development. Considering the similarity in the structure and physiology of inner ear hair cells and retinal photoreceptors between humans and mice, the findings in this report suggest that AC6 may function in the ADGRV1-Gα-PKA signaling pathway at the ankle link complex in inner ear hair cells. However, AC6 does not play an essential role in the development and maintenance of cochlear and retinal structure and function and the pathogenesis of Usher syndrome.

## Methods

### Mice

*Adcy6*^*−/−*^ (also known as *Adcy6*^*tm1Hkh*^) mice and *Ush2a*^*−/−*^ (also known as *Ush2a*^*tm1Tili*^) mice were generated and characterized previously^23,50^. In these mice, exon 1 of the *Adcy6* gene and exon 5 of the *Ush2a* gene were replaced by a Neo^r^ expression cassette. *Adgrv1*^*−/−*^ (also known as *Adgrv1*^*frings*^) mice carried a naturally occurring mutation c.6864delG in the *Adgrv1* gene. The *Adgrv1*^*−/−*^ mice were obtained by crossing BUB/BnJ mice (Jax stock#000653) and wild-type mice with a mixed genetic background of C57BL/6 and 129sv to eliminate the *Pde6b*^*rd1*^ mutation. The inner ear phenotype of the *Adgrv1*^*−/−*^ mice was characterized previously^30^. *Pde6b*^*rd1*^ mice (referred to as *Rd1* here) had a naturally occurring *Pde6b* mutant allele c.1041C>A and were free of *Adgrv1* mutation. The *Rd1* mice were generated during the process of *Adgrv1*^*−/−*^ mouse generation. All experiments involving animals were performed in compliance with animal protocol 20-03001 approved by the Institutional Animal Care and Use Committee at the University of Utah. For terminal experiments, mice were euthanized by CO_2_ inhalation, consistent with the recommendations of the Panel on Euthanasia of the American Veterinary Medical Association (AVMA). The guidelines of ARRIVE (Animal Research: Reporting of In Vivo Experiments) were followed.

### Antibodies

The information on the primary antibody purchase, validation, and usage is provided in Table 1. Alexa Fluor 488-conjuated phalloidin, Alexa Fluor 594-conjuated secondary antibodies, and Hoechst 33342 dye were bought from ThermoFisher Scientific (Waltham, MA, United States). Horseradish peroxidase-conjugated secondary antibodies were purchased from Jackson ImmunoResearch (West Grove, PA, United States).

**Table 1:** Primary antibodies used in this study.

Name	Cat #	Company	Host & Type	Validation	Concentration/dilution ratio^c^
ADGRV1^a^	N/A	N/A	Rabbit polyclonal	Immunostaining in *Adgrv1*^*−/−*^ inner ears^30^	IF: 1:4000
AC5/6	sc-590	Santa Cruz	Rabbit polyclonal (C-17)	Immunostaining in *Adcy6*^*−/−*^ inner ears (this study)	IF: 2 mg/ml WB: 0.2 mg/ml
Gαi2	sc-13534	Santa Cruz	Mouse monoclonal (L5)	Immunoblot analysis in transfected HEK293 cells (this study)	IF: 2 mg/ml WB: 0.2 mg/ml
Gαs	sc-823	Santa Cruz	Rabbit polyclonal (K-20)	Immunoblot analysis in transfected HEK293 cells (this study)	IF: 2 mg/ml WB: 0.2 mg/ml
γ-actin	sc-65634	Scant Cruz	Mouse monoclonal (2–4)	Immunostaining and immunoblot analyses in *Actg1*^*−/−*^ tissues^57^	WB: 0.1 mg/ml
Pan-PKA Cα	4782	Cell signaling	Rabbit polyclonal	Immunoblot analysis in siRNA knockdown Hela cells (by seller)	IF: 1:100 WB: 1:1,000
Phospho-PKA Cα (Thr198)^b^	4781	Cell signaling	Rabbit polyclonal	Immunoblot analysis of phosphatase-treated C6 and 3T3 cells (by seller)	IF: 1:100 WB: 1:1,000
Pan-Akt	4691	Cell signaling	Rabbit monoclonal (C67E7)	Immunostaining of insulin-treated C2C12 cells and immunoblot analysis of recombinant Akt proteins (by seller)	IF: 1:100 WB: 1:1,000
Phospho-Akt (Ser473)	4060	Cell signaling	Rabbit monoclonal (D9E)	Immunostaining and immunoblot analyses of various LY294002-treated and PDGF-treated cultured cells; Immunoprecipitation of Jurkat extracts using non-specific and specific antibodies (by seller)	IF: 1:100 WB: 1:1,000
FLAG tag	F1804	Sigma	Mouse monoclonal (M2)	Immunoblot analysis in transfected HEK293 cells (this study)	WB: 0.3 mg/ml
γ-tubulin	T6557	Sigma	Mouse monoclonal (GTU-88)	Immunoblot analysis in cultured cells by skipping the primary antibody (by seller)	WB: 1:10,000

^a^This antibody was generated in our laboratory^30^.

^b^The position of phosphorylated threonine in PKA Cα was changed from 197, indicated by Cell Signaling, to 198, based on the human and mouse PRKACA isoform 1 sequences NP_002721 and NP_032880, respectively, on NCBI website.

^c^Dilution ratios were provided when the antibody concentrations were unavailable by the selling company.

### Reverse transcription (RT), polymerase chain reaction (PCR), cDNA cloning and transfection

Total RNA was isolated from adult mouse retinas using TRIzol Reagent (ThermoFisher Scientific, Waltham, MA, United States). RT and PCR were performed using ThermoScript RT-PCR system (ThermoFisher Scientific, Waltham, MA, United States) and Expand Long Template PCR system (Roche Diagnostics, Indianapolis, IN, United States), respectively, according to the manufacturers’ instructions. PCR conditions were 95 °C for 30 s followed by 35 cycles of 61 °C (AC1-AC10 genes) or 60 °C (Gα genes) for 30 s and 72 °C for 1 min. Primer information is listed in Table 2. The final RT-PCR products were subjected to agarose gel electrophoresis and ethidium bromide staining. For the AC gene expressions, DNA signals on the agarose gel were imaged using the AlphaInnotech AlphaView software by a FluorChem Q machine (Cell Biosciences, Inc., Santa Clara, CA, United States). The Gα protein cDNAs were cloned into the p3xFLAG-Myc-CMV-25 plasmid (Sigma Aldrich, St. Louis, MO, United States) in fusion with the preprotrypsin leader sequence and 3XFLAG tag at the NotI and KpnI sites and were verified by DNA sequencing. HEK293 cells were cultured in Dulbecco’s modified Eagle’s medium supplemented with 5% fetal bovine serum and 1% penicillin–streptomycin (ThermoFisher Scientific, Waltham, MA, United States). Transient transfection was conducted using Lipofectamin 2000 transfection reagent (ThermoFisher Scientific, Waltham, MA, United States). Cell lysates were analyzed at ~ 24 h after transfection.

**Table 2:** Primer information for RT-PCR.

Gene	Product region	Forward	Reverse
AC1	11,107–11,706 bp (NM_009622)	CAACGGACATGCTATCTGAG	CCACTGCACAGTAAGTGCTG
AC2	3401–4000 bp (NM_153534)	TCATCCTGCAGACGCTTGGC	GCAATGAGACACACGGGTGG
AC3	3551–4120 bp (NM_138305)	AGCTGCCTTCCCCAATGGCT	TCTGCTCTCGTGACTAAGGT
AC4	2754–3313 bp (NM_080435)	TGAGGACCTCTACCACCAGT	TGCCTCTGCTATAGCACGTG
AC5	5093–5632 bp (NM_001012765)	CAGATAGGGCCTGTGCTCCA	TGCTCAGAGCCACTGCTCCT
AC6	5301–5840 bp (NM_007405)	CTGTCCTCGCCTGCCCTTGC	ACATGACTGGCTGGCACTGT
AC7	5321–5850 bp (NM_007406)	AAGGCTGAGTAGTCAGGGAC	AACGAATCCTTGACCCACAG
AC8	4301–4860 bp (NM_009623)	CATACATGGCTGTCTCAGGA	GTAATGGCTCTTGATGATGC
AC9	4425–5004 bp (NM_009624)	GTTATGACTTTGACTACCGA	AATGGGTGGAAATGAACCCT
AC10	4601–5160 bp (NM_173029)	CTAGGTACATGGAAGGGCAA	ATGGCTCCGGAAGCTGGCAC
GNAI2	130–1194 bp (NM_008138)	gcgccgcgGGCTGCACCGTGAGCGCC	ggtaccTCAGAAGAGGCCACAGTCCTTC
GNAS	294–1475 bp (NM_201616)	gcggccgcgGGCTGCCTCGGCAACAGTA	ggtaccTTAGAGCAGCTCGTATTGGC
GNAQ	646–1722 bp (NM_008139)	gcggccgcgACTCTGGAGTCCATCATGG	ggtaccTTAGACCAGATTGTACTCCTTC

The sequences in lower case letters are sequences of restriction enzyme sites.

### Immunostaining and immunoblot analyses

Immunostaining procedures were similar to those described previously^51–53^. Briefly, mouse temporal bones at P4 were isolated and fixed in 4% formaldehyde/phosphate-buffered saline (PBS) for about 10 min. Cochleas and vestibular organs were then dissected and fixed in 4% formaldehyde/PBS for 30–120 min depending on the antibodies used. The fixed inner ear tissues were permeabilized in 0.5% Triton X-100/PBS for 15–20 min. Mouse eyes were enucleated and frozen immediately in Tissue-Tek OCT compound on dry ice. The retinas were then sectioned at 10 µm, fixed in 4% formaldehyde/PBS for 10 min, and permeabilized in 0.2% Triton X-100/PBS for 5 min.

The whole mount inner ear tissues or retinal sections were blocked in 5% goat serum/PBS for 1 h, then incubated with primary antibodies in blocking solution at 4 °C overnight. The dilution ratios of the primary antibodies were determined according to the manufacturers’ instructions (Table 1). After extensive washes with PBS, the tissues or sections were incubated with Alexa fluorochrome-conjugated secondary antibodies in blocking solution for 1 h. Fluorescent images were taken using a confocal laser scanning microscope (Model FV1000, 60X UPLSAPO objective, numerical aperture: 1.42, Olympus, Tokyo, Japan or Model SP8 Lightning Super-Resolution, 63X HCPLAPO objective, numerical aperture: 1.4, Leica Microsystems, Chicago, IL, United States).

To conduct immunoblot analysis, cochleas or vestibular organs of at least 4 P4 pups were pooled and homogenized in lysis buffer. The recipe of the lysis buffer was 50 mM Tris–HCl, pH 8.0, 150 mM NaCl, 0.5% Triton X-100, 5 mM ethylenediaminetetraacetic acid (EDTA), 0.5 mM phenylmethylsulfonyl fluoride, 1× protease inhibitor, and 1 mM dithiothreitol. The lysates were then cleared by centrifugation at 21,000*g* for 10 min and subjected to the same immunoblotting procedure described in our previous publication^54^. Pan-proteins and phospho-proteins were analyzed either on two separate immunoblots or on the same immunoblots, because the signals from phospho-protein antibodies were extremely lower than those from pan-protein antibodies. γ-actin or γ-tubulin signals were used to assess the changes of pan-protein expressions between genotypes or as loading controls when pan- and phospho-proteins were analyzed on separate immunoblots. To quantify the immunoblot signals, Gels function under the Analyze menu in ImageJ 1.53a (http://imagej.nih.gov/ij, NIH, United States) was utilized.

### Auditory brainstem response (ABR) and distortion product otoacoustic emission (DPOAE) tests

ABR and DPOAE procedures were performed as described previously^53,55^. Mice were anesthetized by intraperitoneal injection of ketamine (100 mg/kg) and xylazine (10 mg/kg). Stimuli for ABR tests were generated digitally in SigGenRP through an electrostatic speaker (EC-1, Tucker-Davis Technology, Alachua, FL, United States). Recording electrodes were placed under the skin at the vertex and mastoid, and a ground electrode was placed in the rump area. ABR thresholds were determined as the lowest sound pressure levels (SPLs) at which the response was clearly discernible. Stimuli for DPOAE tests were generated digitally through an ER10B + microphone (Etymotic Research, Elk Gove Village, IL, United States) coupled with two EC-1 speakers. Stimuli of two primary tone frequencies f_1_ and f_2_ (f_2_/f_1_ = 1.2) were presented with L_2_ = L_1_ − 10 dB. The ear canal sound pressure was recorded and processed. DPOAE thresholds were determined as the lowest L_1_ SPLs at which the 2f1-f2 distortion product was observable above the noise floor. The ABR and DPOAE tests were conducted by two people who were blind to the genotype. The data generated by the two people were combined before the statistical analysis.

### Electroretinogram (ERG) test

ERG tests were conducted using a UTAS-E3000 system (LKC Technologies, Gaithersburg, MD, United States)^56^. Mice were first dark adapted overnight. After anesthesia by intraperitoneal injection of ketamine (100 mg/kg) and xylazine (10 mg/kg), pupils were dilated with 1% tropicamide. A recording electrode was placed on the cornea, and a subdermal reference electrode was placed around the test eye. Scotopic ERGs were recorded at flashes of full-field white light in darkness. After 35 cds/m^2^ background illumination for 10 min, photopic ERGs were recorded at flashes of full-field white flashes in the presence of the same background illumination. A-wave and b-wave amplitudes were measured from the difference between the baseline and the cornea-negative peak and the difference between the cornea-negative peak and the major cornea-positive peak, respectively. A-wave and b-wave implicit times were measured from the onset of stimuli to the cornea-negative peak and to the major cornea-positive peak, respectively. The ERG tests were conducted by two people who were blind to the genotype. The data generated by the two people were combined before the statistical analysis.

### Statistical analyses

Statistical analyses were performed using Graphpad Prism 9 (macOS, version 9.5.1, http://www.graphpad.com). Repeated measures two-way ANOVA with genotype as a between-subject variable and tone frequency or light intensity as a within-subject variable was used to analyze the genotype effect on ABR, DPOAE, or ERG responses. When ABR and ERG values were missing at some tone frequencies or light intensities, mixed-effects model analysis was conducted instead. Dunnett’s multiple comparisons test was conducted to calculate the adjusted P values while comparing data of *Adcy6*^+*/−*^ and *Adcy6*^*−/−*^ mice with those of wild-type mice. Nonparametric Mann–Whitney test was conducted to compare phospho-protein expression in cochlear and vestibular organs between *Adcy6*^*−/−*^ and wild-type genotypes. P values and adjusted P values smaller than 0.05 were considered to indicate a significant difference.

## Supplementary Information

Supplementary Information.

## Data Availability

The data generated and analyzed in the current study are available from the corresponding author upon reasonable request.
