# Supplementary material for: Adenylyl cyclase 6 plays a minor role in the mouse inner ear and retina
Source: Sci Rep. 2023 May 1;13:7075. doi: 10.1038/s41598-023-34361-y (PMC10151359; doi:10.1038/s41598-023-34361-y)
Supplement: Supplementary file 1 — Supplementary Information. [file 41598_2023_34361_MOESM1_ESM.pdf]

Adenylyl cyclase 6 plays a minor role in the mouse inner ear and retina

Pranav Dinesh Mathur[1,2#†], Junhuang Zou[1†], Grace Neiswanger[1], Daniel Zhu[1], Yong Wang[3], Ali A. Almishaal[4,5], Deepti Vashist[1], H. Kirk Hammond[6], Albert H. Park[3], Jun Yang[1,2,3*]

Supplementary Figure 1: AC6 stereociliary localization in wild-type, *Adgrv1$^{-/-}$*, and *Ush2a$^{-/-}$* cochlear hair cells. (a) AC6 (magenta) is localized to the basal portion of stereocilia stained by phalloidin (green) in wild-type cochlear IHCs and OHCs at P4. **(b)** AC6 immunostaining signal is lost in *Adcy6$^{-/-}$* IHCs and OHCs at P4. **(c)** ADGRV1 (magenta) labels the ankle link complex in cochlear IHCs and OHCs at P4. **(d** and **e)** The distribution of AC6 signal is extended to the entire stereocilia in IHCs and OHCs of *Adgrv1$^{-/-}$* **(d)** and *Ush2a$^{-/-}$* **(e)** mice at P4. The merged images on the right are the corresponding view of the magenta-colored images on the left. Scale bars: 2 µm.

Supplementary Figure 2: Gα and PKA localization in cochlear hair cell stereocilia. (a) Immunostaining shows that Gαs protein (magenta) is located along the entire stereocilia (green) except the tip and base in wild-type cochlear hair cells at P4. **(b)** Immunostaining shows that Gαi protein (magenta) is located at the very tip and around the ankle link complex region in wild-type cochlear stereocilia at P4. **(c** and **d)** Immunostaining shows that pan-PKA Cα1 (PKA, magenta, **c**) and phospho-PKA Cα1 (pPKA, magenta, **d**) are located at the basal portion of stereocilia in wild-type IHCs and OHCs at P4. **(e)** The distribution of phospho-PKA Cα1 subunit in *Adcy6$^{-/-}$* cochlear hair cells at P4 is similar to that in wild-type cochlear hair cells in **d**. The merged images (right) correspond to the single-channel magenta images (left). Scale bars: 2 µm.

Supplementary Figure 3: Normal pan- and phospho-Akt localizations in *Adcy6$^{-/-}$* cochleas. Immunoreactivities of pan-Akt (magenta, **a** and **b**) and phospho-Akt (pAkt, magenta, **c** and **d**) are detected in the stereociliary bundle (green) and cell body of cochlear hair cells as well as surrounding cells in wild-type (**a** and **c**) and *Adcy6$^{-/-}$* (**b** and **d**) littermate mice at P4. There are no obvious differences in the pan-Akt and phospho-Akt expression patterns between wild-type and *Adcy6$^{-/-}$* mice. The merged images (right) correspond to the single-channel magenta images (left). Scale bars: 2 µm.

Supplementary Figure 4: Original immunoblot and DNA gel images presented in the main manuscript. The panel labels are the same as in the main figures. The additional bands in the Gαs blot (Figure 2a) are from the endogenous Gαs proteins from HEK293 cells.

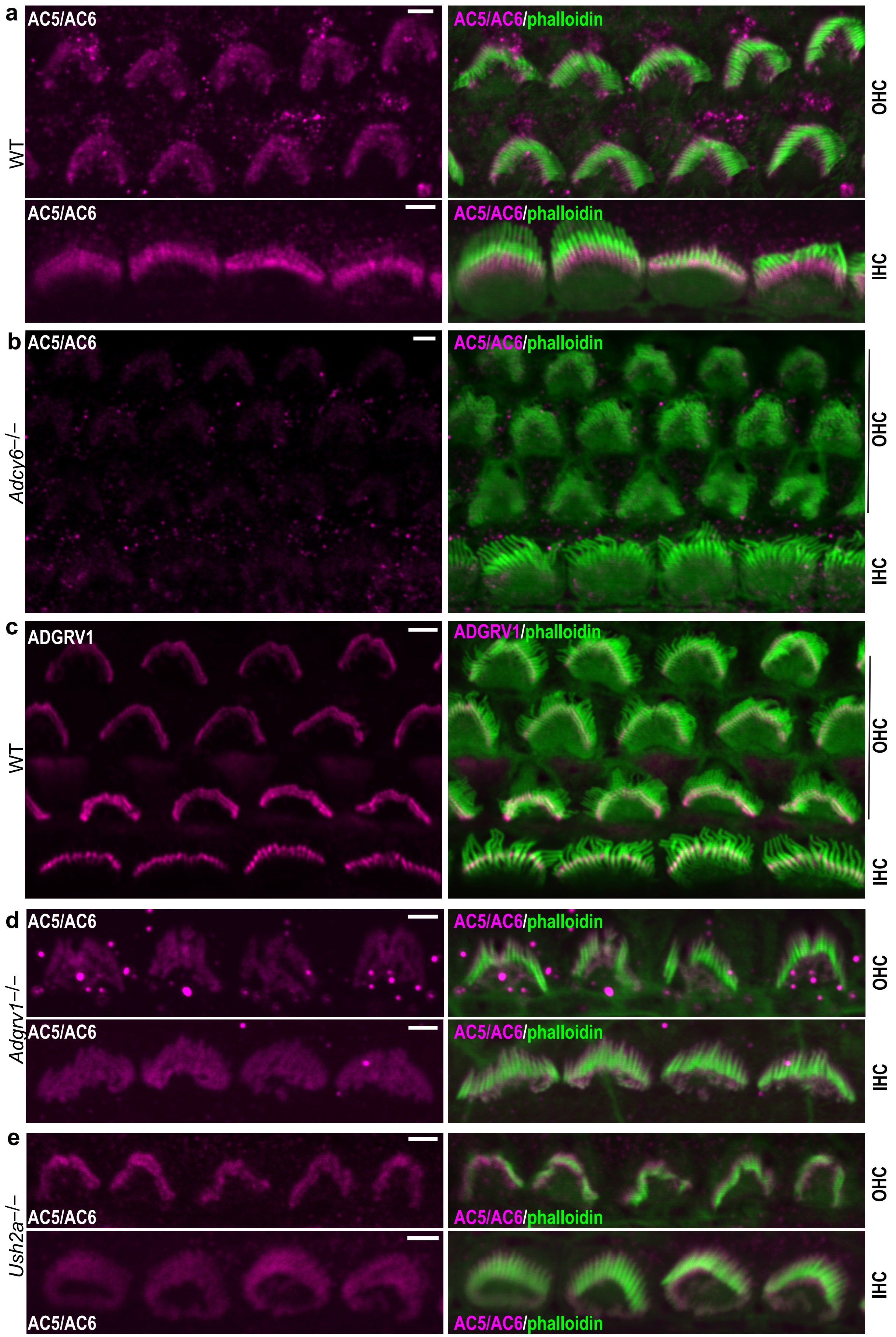

a
AC5/AC6
AC5/AC6/phalloidin
WT
OHC
AC5/AC6
AC5/AC6/phalloidin
IHC
b
AC5/AC6
AC5/AC6/phalloidin
Adcy6−/−
OHC
IHC
c
ADGRV1
ADGRV1/phalloidin
WT
OHC
IHC
d
AC5/AC6
AC5/AC6/phalloidin
Adgrv1−/−
OHC
AC5/AC6
AC5/AC6/phalloidin
IHC
e
Ush2a−/−
AC5/AC6
AC5/AC6/phalloidin
OHC
AC5/AC6
AC5/AC6/phalloidin
IHC

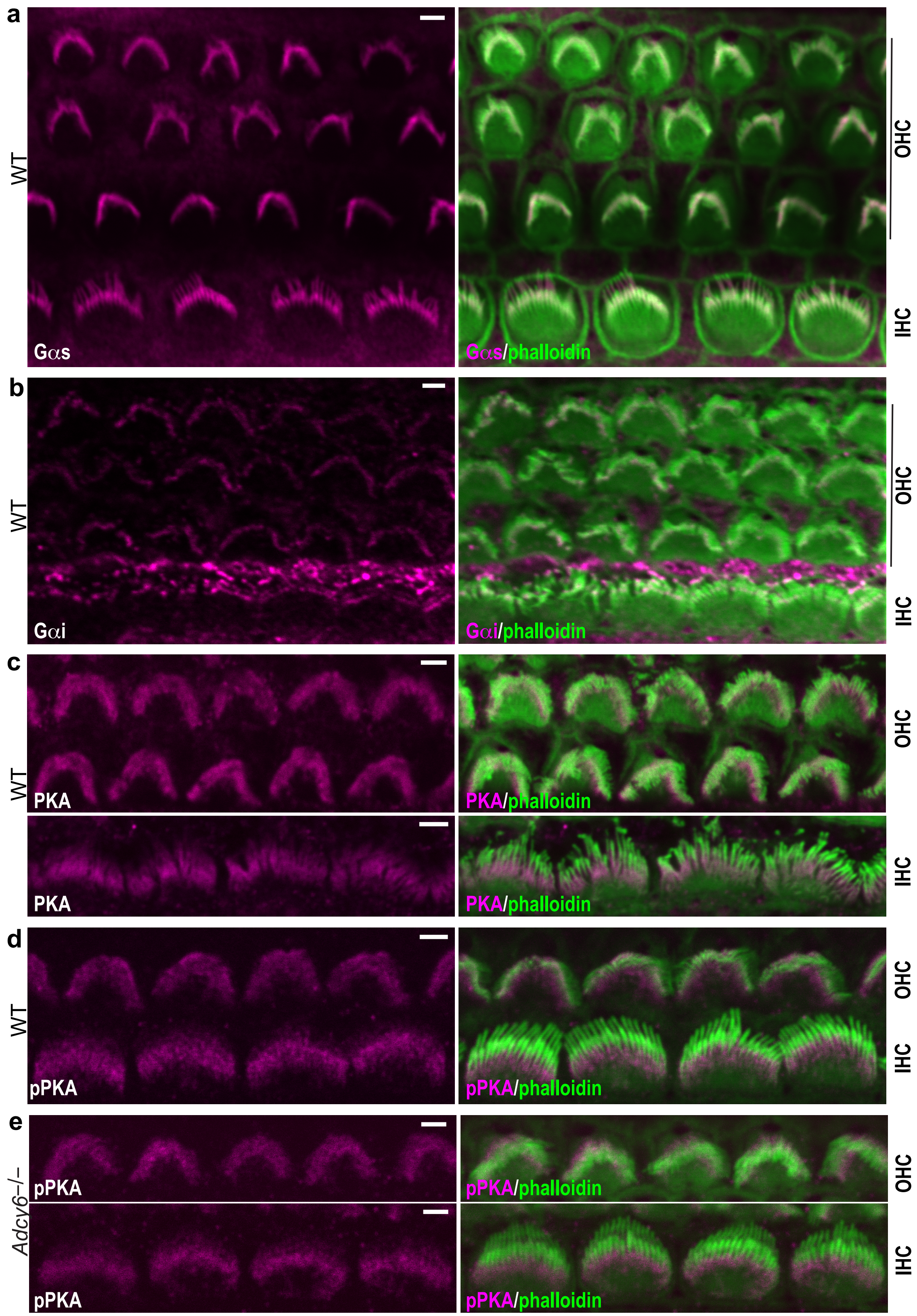
a
WT
Gαs
Gαs/phalloidin
OHC
IHC
b
WT
Gαi
Gαi/phalloidin
OHC
IHC
c
WT
PKA
PKA/phalloidin
OHC
PKA
PKA/phalloidin
IHC
d
WT
pPKA
pPKA/phalloidin
OHC
IHC
e
Adcy6−/−
pPKA
pPKA/phalloidin
OHC
pPKA
pPKA/phalloidin
IHC

Figure S2

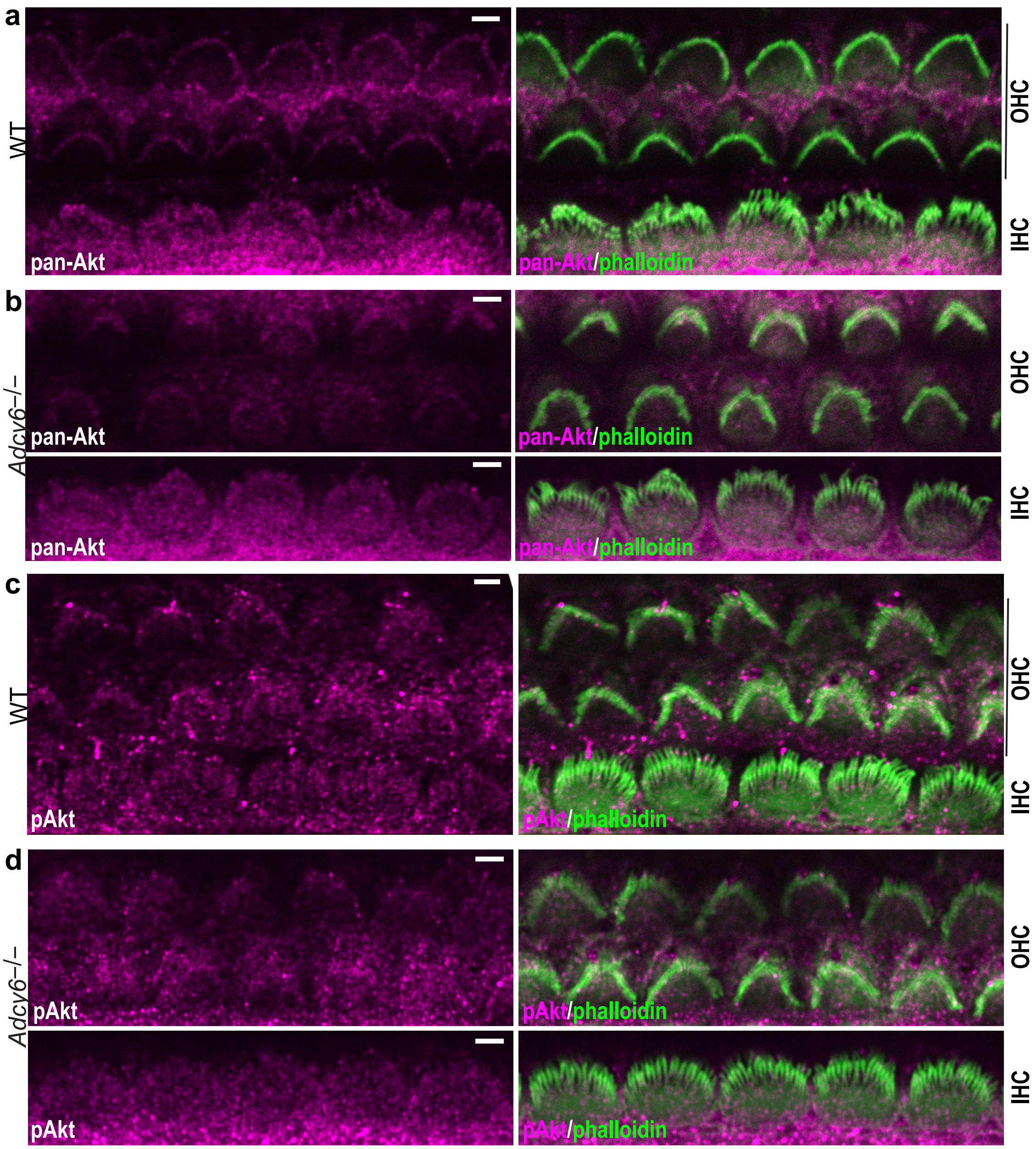

a
WT
pan-Akt
pan-Akt/phalloidin
OHC
IHC
b
Adcy6−/−
pan-Akt
pan-Akt/phalloidin
OHC
pan-Akt
pan-Akt/phalloidin
IHC
c
WT
pAkt
pAkt/phalloidin
OHC
IHC
d
Adcy6−/−
pAkt
pAkt/phalloidin
OHC
pAkt
pAkt/phalloidin
IHC

Figure S3

Figure 2a

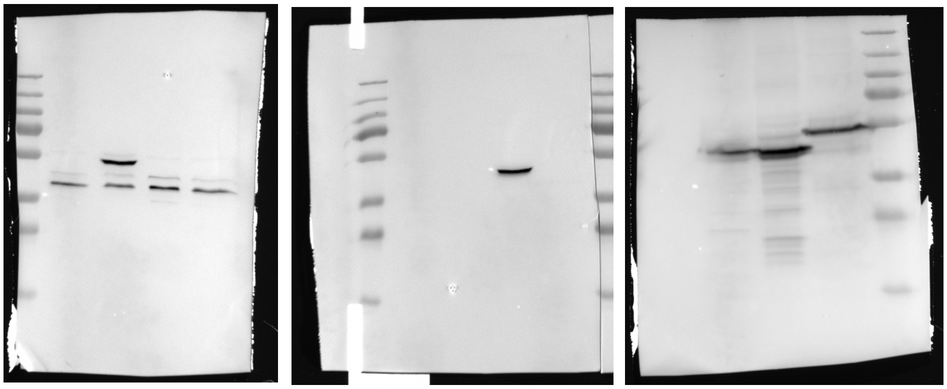

Gαs Gαi FLAG

Figure 2e

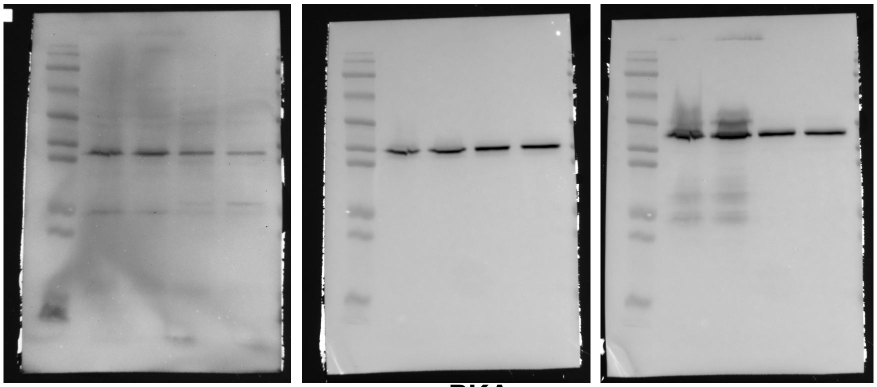

pPKA pan-PKA γ-tubulin

Figure 3c

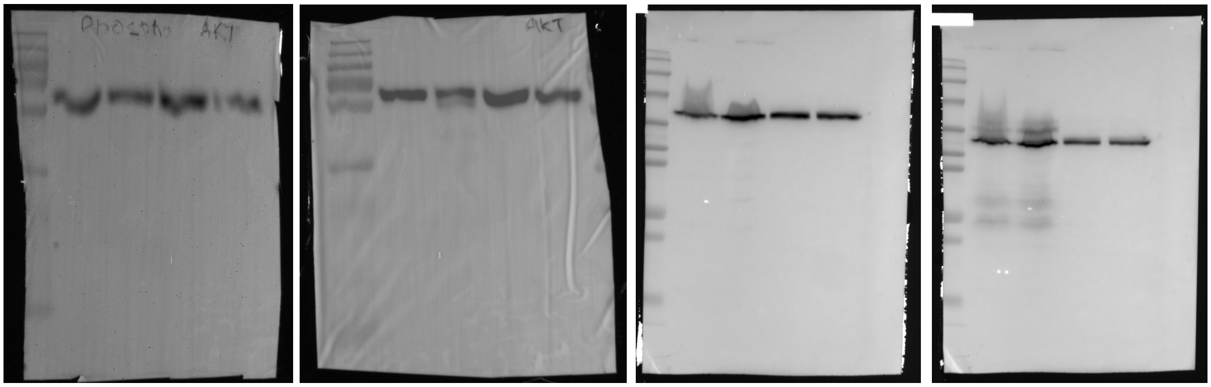

pAkt pan-Akt pan-Akt γ-tubulin

Figure 5a

AC RT-PCR

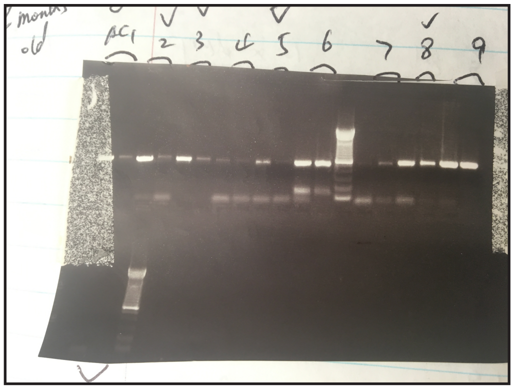

Figure S4
